# Effect of Hammer Type on Generated Mechanical Signals in Impact-Echo Testing

**DOI:** 10.3390/ma14030606

**Published:** 2021-01-28

**Authors:** Richard Dvořák, Libor Topolář

**Affiliations:** Faculty of Civil Engineering, Brno University of Technology, 602 00 Brno, Czech Republic; Libor.Toplar@vutbr.cz

**Keywords:** impact-echo method, hammer, fast Fourier transform, Saaty matrix, feature extraction, nondestructive testing, civil engineering

## Abstract

The impact-echo diagnostic method is a well-known nondestructive pulse compression test method, which can be relatively easily used for the testing of concrete and reinforced concrete elements. The evaluation of the measurement with this method is based on the analysis of the signal itself in the time and frequency domains. This allows acquisition of information on the velocity of the mechanical wave, the resonant frequency of the specimen or on the presence of internal defects. The ability to interpret these measurements depends on the experience of the diagnostic technician. The advent of classification algorithms in the field of machine learning has brought an increasing number of applications where the entire interpretation phase can be considerably simplified with the help of classification models. However, this automated evaluation procedure must be provided with the information of whether the signal acquired by the test equipment has actually been measured under optimally set conditions. This paper proposes a procedure for the mutual comparison of different measuring setups with a variable tip type, hammer handle and impact force. These three variables were used for a series of measurements which were subsequently compared with each other using multi-criteria evaluation. This offers a tool for the evaluation of measured data and their filtering. As an output of the designed method, each measurement is marked by a score value, which represents how well the acquired signal fit the weight demands for each observed feature of the signal. The method allows the adjustment of selected demands for a specific application by means of set thresholds. This approach enables the understanding of characteristics of the signal in the automated pre-processing of measured data, where computing power is limited. Thus, this solution is potentially suitable for remote long-term observations with sensor arrays or for acoustic emission signals pre-processing.

## 1. Introduction

Testing of the thickness of concrete elements in the 1990s began to include the acoustic nondestructive impact-echo method [1,2,3], also called the resonance method [4], with a mechanical impulse generated by a hammer (sometimes also the hammer method [5]). The impact-echo (IE) method has found wide application in the construction industry due to its simplicity, low implementation cost and a relatively wide range of possible uses. However, this method is also dependant on the correct interpretation of measured data. In practice, it is employed to measure the length of piles, to localise cracks in massive monolithic structures, to detect and localise the delamination of bridge decks, to diagnose the condition of concrete elements, etc. [6,7,8,9]. In the preservation of cultural heritage, it is also used either as an in-situ testing method of the current state of structural elements or as a monitoring tool in the form of a sensor array [10,11,12]. Due to a simple testing principle, there are many variations of this method in the form of, for example, the low-frequency pulse-echo method [13,14] or modal analysis [15,16,17].

Knocking on a structure with a hammer is the oldest method of nondestructive testing in construction. It is based on the propagation of a mechanical wave through a structure. Depending on the sound (in the hearing range of 20 Hz–20 kHz) produced by the knocking, whether it is a high, clearly audible sound or a deep and muffled sound, it is possible to assume the state of the structure and whether or not it has any defects. The method (when used within the hearing range) is subjective and depends on experience and surrounding conditions of such testing.

The IE method plays an important role in the nondestructive testing of civil engineering structures, namely concrete structures. Materials, such as concrete, or ceramic-based materials and composites are strongly heterogeneous compared to steel elements preferably used in mechanical and aeronautics engineering. In past years, many non-destructive (NDT) methods have been developed, which give satisfactory and accurate results in testing metal alloys [18,19,20] but cannot be used with the same effectiveness in testing building materials. The main problem lies in refracting mechanical waves from natural defects and morphological elements, such as air voids and different types of aggregate and cracks caused by cement shrinkage. All these built-in defects are considered part of a healthy structure from the point of view of technical norms and standards used in civil engineering. Nondestructive standards are closer to mechanical applications, and usage of NDT acoustic techniques in building materials requires some precaution. Pulse echo signals acquired from testing such heterogeneous materials naturally contain more noise, due to the porous character, and the material slightly changes its acoustic impedance with different moisture content [21]. What can be measured in homogenous steel elements cannot be measured with the same accuracy and reliability in concrete elements, due to material and physics limitations.

The impact-echo method is easy to use and does not require expensive instrumentation. Combining classification models, or deep convolutional neural networks, can be used as a powerful tool seen in this study [22]. Other applications can be found in testing concrete pavements, which is done by a laser crack measurement system [23]. This system allows the inspection of kilometres of highway but can only assess defects present on the top layers of pavement. An array of impactors and microphones as receivers were used in these studies for testing large areas of roads, or bridge decks [24,25] for the presence of voids, and the delaminating of different construction layers.

These studies document the importance of the impact-echo in nondestructive testing in civil engineering as an accessible tool, assessing large structures with relatively low instrumental requirements.

The presented paper extends previous research in the impact-echo method and focuses on the preferable combination of the impact force, handle type and tip shape with regard to the resulting generated signals. It is therefore a methodological analysis of the physical testing procedure itself and simultaneously of the involved instrumental equipment. These experiments included a calibration and comparison of different combinations of signal excitations and their influence on the resulting frequency spectrum.

### 1.1. Mechanical Wave

**Figure 1:**
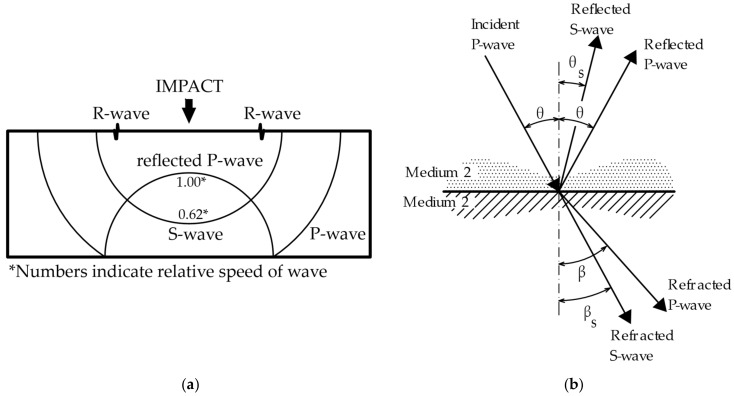
Wave theory: (**a**) Differentiation of waves during signal generation [26]; (**b**) reflection and refraction of waves at the interface of two different materials [30].

Vibrating the surface of a test specimen (from the applied impact stress) generates a mechanical wave through the test medium. Using Figure 1a, we can define three types of mechanical waves that propagate through the material: P-wave, S-wave and R-wave [26]. The P-wave represents longitudinal oscillation associated with tensile and compressive stresses, and achieves the highest velocity compared to the other types of mechanical waves. The S-wave represents transverse oscillation associated with shear stress, and the R-wave is formed by oscillations propagating over the surface of the material, also called Rayleigh surface waves [27]. When mechanical waves travel through a material, the P-wave is fastest and has the highest energy, followed by the S-wave. If the used measuring equipment is set incorrectly, the surface R-waves may be confused with the longitudinal P-waves or the transverse S-waves. This is often caused by placing the receiving sensor close to a hitting position. This may lead to the emergence of frequencies before the resonance frequency itself. Depending on the position of the receiving sensor to hit position, these frequencies may reach even higher amplitudes than the resonance frequency—in such a situation, a false classification will happen, because in most technical norms, the first dominant frequency is assessed (for example, for dynamic modulus of elasticity [28], or thickness of concrete pavement [29]). In measurements up to hundreds of signals, a human operator can still distinguish such an error, but if we want to assess thousands of signals automatically, a serious problem may occur.

The movement of mechanical waves through different types of materials is influenced by their value of acoustic impedance:

Z = ρ × C_p_, (1)

Z—acoustic impedance (kg·m^−2^·s^−1^), ρ—density (kg·m^−3^) and C_p_—velocity of the longitudinal P-wave (m·s^−1^).

Mechanical waves propagate easily through a material that has a high acoustic impedance value (solid materials, e.g., concrete Z = (6.9–10.4) × 10^6^ kg·m^−2·^s^−1^) and is completely absorbed or partially reflected in materials whose acoustic impedance value is close to zero (for example, gaseous substances, air Z = 437 kg·m^−2^·s^−1^). This type of mechanical wave interaction at the material interface is described in Figure 1b. At the moment, when the mechanical wave hits the interface of two materials that have significantly different acoustic impedance values (for example, a cavity containing air inside a concrete mass), mechanical energy is absorbed or partially reflected [27]. This change can be recorded and analysed.

The amplitude of the P-wave reaches the highest values when the angle of incidence of the wave is perpendicular to the interface of two media with different acoustic impedances Z_1_ and Z_2_. In this case, the following equation applies:

R_n_ = (Z_2_ − Z_1_)/(Z_2_ + Z_1_), (2)

R_n_—refractive index for perpendicular incidence, Z_1_—acoustic impedance of the first medium and Z_2_—acoustic impedance of the second medium.

If Z_1_ is higher than Z_2_, the refractive index R is negative. If the incident wave carries compressive stress, the reflected wave will conversely carry tensile stress. If Z_2_ is larger than Z_1_, no change occurs. The refractive index depends on the angle of incidence [27].

In the case of the propagation of mechanical waves through a nonhomogeneous material, mechanical waves are reflected and refracted at each material interface. Each incident P-wave can be refracted, or reflected, again as a P-wave or S-wave.

### 1.2. Impact-Echo Method Principle

**Figure 2:**
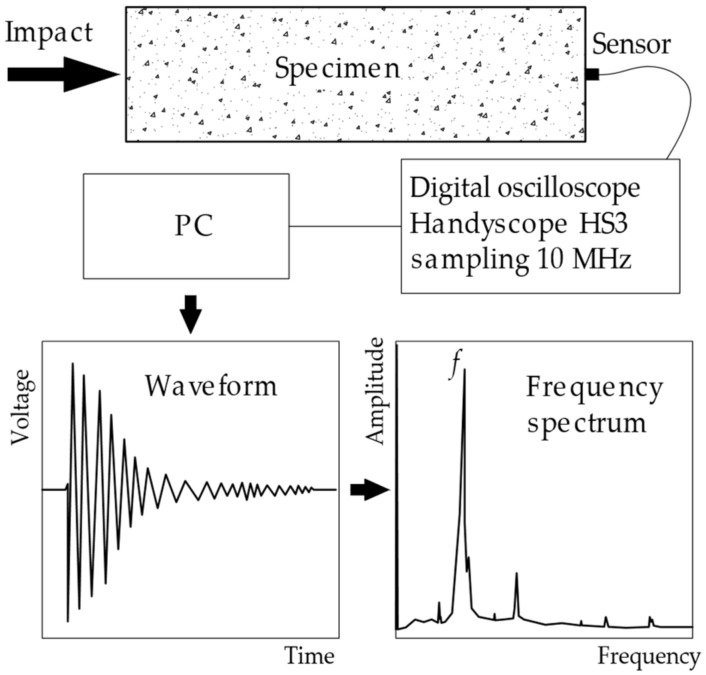
Diagram of the testing procedure using the impact-echo method [27].

The impact-echo method is based on the controlled generation of vibrations using mechanical impact and the subsequent detection of these vibrations from the tested element (the method operates within the range of 3 Hz–20 KHz). The initiation of vibrations in commercially used methods can be triggered by a number of steel hammers, which differ in weight and diameter. Vibration detection is done by piezoelectric sensors, which convert the mechanical wave energy into an analogue signal in the form of voltage U(t). This signal in the time domain is processed using the fast Fourier transform, which can convert the signal to the frequency spectrum U(f). This frequency spectrum can then be a subject of further analysis. The method of spectrum processing differs according to the application on a specific structure and according to the aim of the measurement [30]. The basic composition of the impact-echo measurement is presented in Figure 2.

If we simultaneously record the signal generated by the hammer (acoustic signal generator), we will always receive two signals in any sensor-generator location. The first signal in time is recorded on the hammer and then with a minor delay on the sensor located on the test specimen. This delay is determined by the velocity and type of mechanical wave that is preferred by the generator–sensor orientation. When testing standard laboratory beam test specimens (shown in Figure 2), where the sensor is placed on the front face of the test beam and the impact is directed at the opposite face, the longitudinal P-wave is preferred and the time difference between the two measured signals can be used to derive the velocity of the longitudinal wave C_p_.

On the other hand, if the sensor is placed on the same surface as the point of impact of the hammer, it is the surface (R-wave) and shear (S-wave) waves that are recorded. This difference must be considered when designing and implementing NDT testing using the impact-echo method.

**Figure 3:**
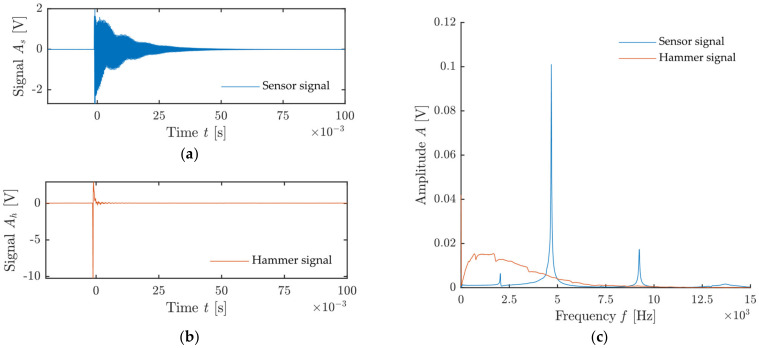
Comparison of sensor and hammer signals and their frequency spectrum: (**a**) Signal of receiving sensor attached to test specimen; (**b**) signal of hammer sensor; (**c**) frequency spectrum of both signals.

An actual comparison of the recorded signals and their frequency spectrum is presented in Figure 3. The left graph shows the hammer signals on the vertical axis on the right and the sensor signal on the vertical axis on the left. As can be observed, the first amplitude (V) of the hammer signal is 5× higher than the signal recorded by the sensor attached to the test specimen. The right graph shows the frequency spectra of both recorded signals. It is apparent that the dominant frequency of the test specimen is 5 kHz.

### 1.3. Software Equipment and Programs

In the field of vibroacoustics, measured data are processed exclusively by software, either provided directly by the manufacturer of the measuring apparatus or using licensed or open-source libraries and toolboxes. In the case of this application, the limiting factor is often the software license for which the manufacturer clearly defines its use. This creates a situation where the measuring apparatus frequently requires the additional purchase of similarly expensive software.

On the other hand, other methods can be implemented with the use of basic laboratory equipment and can very well provide results equivalent to turnkey commercial systems. This example can be demonstrated on the impact-echo method, where individual manufacturers offer sophisticated measuring instruments with mechanical impact generators together with evaluation software.

This type of apparatus can be adequately replaced by relatively less expensive stand-alone sensors—for example, by multi-channel digital oscilloscopes, which today provide a sufficient range of recorded frequencies, bit resolution and sufficient data flow, and are therefore capable of the continuous recording of even minute-long signals on the order of MHz. The subsequent appropriate processing of the measured signals is then a matter of selecting the suitable software that includes the necessary functions.

### 1.4. Fast Fourier Transform

Acoustic NDT methods often work with measured signals that consist of a change in voltage U over time t. They can be divided into stationary or ergodic signals. These signals can be expressed in many different ways; time and frequency representations are important. In the Fourier transform, the signal is compared to a complex sine function, and because it is performed over the entire time representation, the frequency spectrum is independent of time. The analysis of such a frequency spectrum is one of the standardised and useful tools for signal studying.

The discrete Fourier transform, inverse Fourier transform or fast Fourier transform, is often discussed in the case of such processing. It is the most frequently used one in practice due to its lower computing demands.

The fast Fourier transform (FFT) algorithm was developed in 1965. The fast Fourier transform [30] can be expressed as follows *f*(*t*):

(3) $$F\left(\omega \right)=\frac{1}{\sqrt{2\pi }}\overset{\infty }{\underset{-\infty }{\int }}f\left(t\right)e^{-i\omega t}dt$$

This transformation allows decomposition of the signal into individual frequencies that comprise the signal. This is an approximate estimate of individual frequencies ω of the short time interval of the signal *t*_0_.

**Figure 4:**
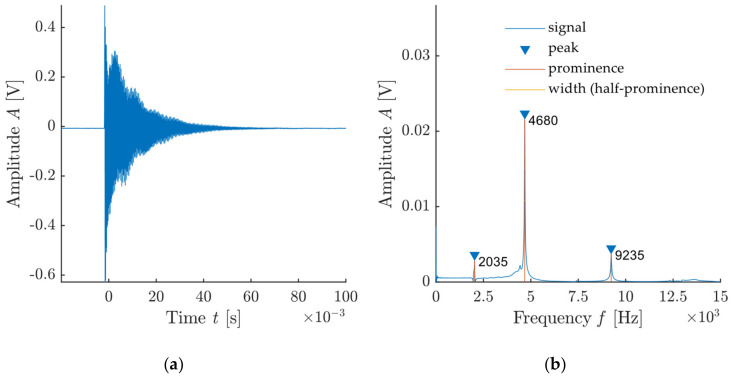
Example of a signal and its frequency spectrum created by FFT: (**a**) Signal from the sensor attached to test specimen; (**b**) frequency spectrum of recorded signal with highlighted dominant frequencies and its parameters.

An example of a signal recorded during impact-echo measurement and its frequency spectrum created by FFT are presented in Figure 4. This graph also documents the typical pulse signal in audible frequencies where signal attenuation occurs.

The resulting frequency spectrum can then be further evaluated and analysed. This procedure is one of the most common tools of analysing signals from the impact-echo method, as is documented by the list of foreign publications and standards [26,29,31,32,33]. The first dominant frequency, or its other harmonic frequencies, is the most frequently evaluated element. The presence of defects, cracks, cavities or another material interface with a significantly different acoustic impedance will influence the resulting frequency spectrum, by shifting the dominant frequencies to higher or lower regions.

## 2. Equipment and Software Used

**Figure 5:**
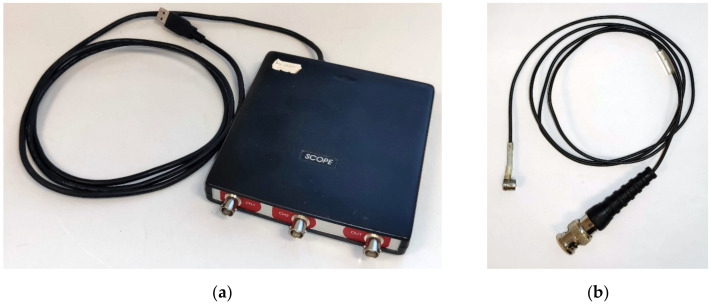
Photos: (**a**) Three-channel digital oscilloscope; (**b**) MIDI-type piezoelectric sensor.

A Handyscope digital oscilloscope was used in the experiment and offers an optimal tool for simple calibrations of even complex measurement procedures, such as modal analysis, due to the relative simplicity of the instrumental part, as well as rich variability of the programming part. The digital oscilloscope Handyscope HS3AWG-5 has a resolution of 16 bits and a maximum bus frequency of 195 kHz at the given resolution. This oscilloscope is connected via a USB connector and can also be used as a multimeter or a signal generator (see Figure 5a). A MIDI 446s12-type piezoelectric sensor supplied by ZD Rpety-Dakel was used to record the generated signal (Figure 5b).

### 2.1. Hammer with a Piezoelectric Sensor

**Figure 6:**
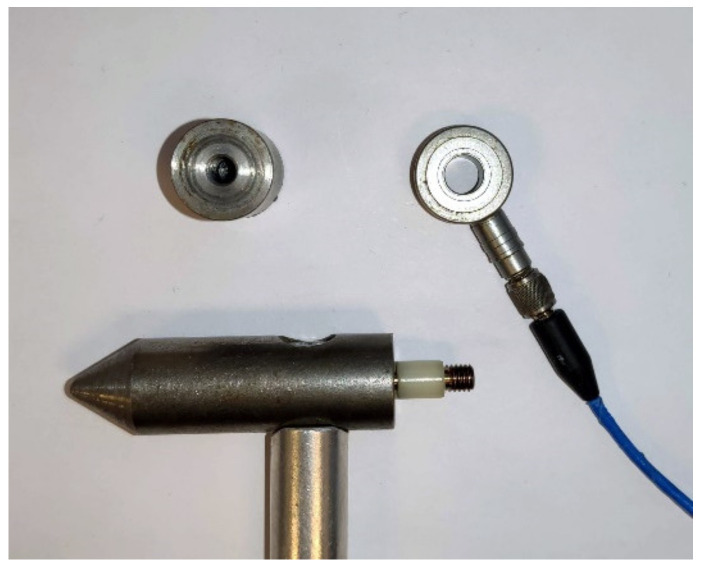
Circular sensor with detail of the mounting in the hammer.

**Figure 7:**
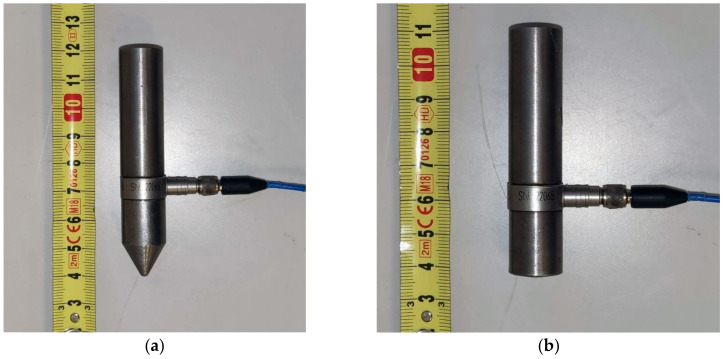
Hammer without a handle: (**a**) With a blunt tip; (**b**) with a sharp tip.

**Figure 8:**
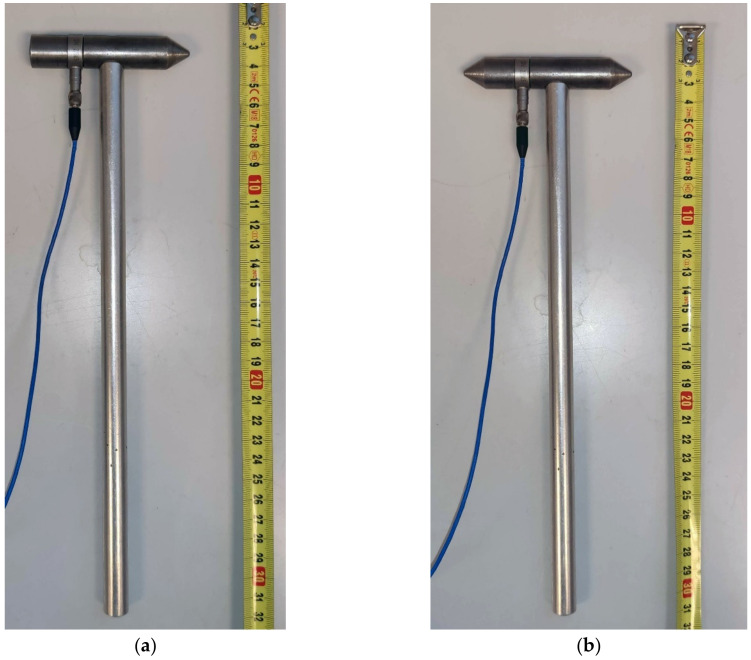
Hammer with a handle: (**a**) With a blunt tip; (**b**) with a sharp tip.

**Figure 9:**
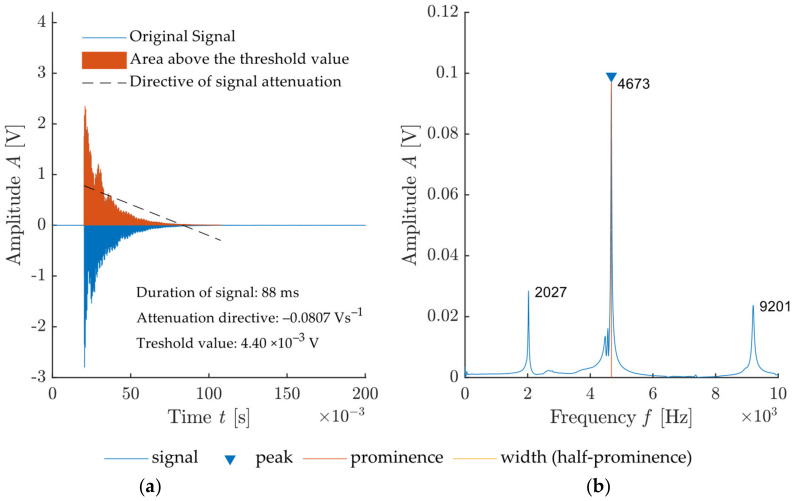
Illustration of all extracted features in the signal by a proposed algorithm: (**a**) Recorded signal from a sensor attached to test specimen with highlighted features (threshold value, signal energy—area above the threshold value, the directive of signal attenuation, signal length); (**b**) frequency spectrum of recorded signal with highlighted frequency features (dominant frequency, the amplitude of dominant frequency, prominence and peak-width at half-prominence).

The experiment was conducted with a hammer with a built-in generator in various configurations, as can be seen in Figure 5, Figure 6, Figure 7, Figure 8 and Figure 9. Unlike a classic hammer for the impact-echo method, the given hammer can record the energy of the generated pulse and the energy of the response of the measured system to the generated signal.

The calibration involved two tip variants and two variants with/without the handle. The first tip variant was a blunt tip with a radius of curvature of 300 mm. This tip can be seen in Figure 7a in combination without the handle and Figure 8a in combination with the handle. The second tip variant was a sharp tip. This tip can be seen in both combinations in Figure 7b and Figure 8b. Calibration measurements were conducted on the test specimen using both variants.

For this calibration, the sensor was placed in the longitudinal frequency *f_L_* measurement orientation. Several impacts were performed until the movement of the specimen on the mat started to enter the measured signal. This situation is already beyond the distortion of the measured signal of the test specimen, and the typical generating mechanical impulse is significantly lower; however, the aim of the calibration was to test the used instruments under various conditions and therefore also included the disproportionate intensity of the hammer impact. The measurement recorded both sensor and generator-hammer signals.

### 2.2. Feature Extraction

Over the course of the measurement with the acoustic impact-echo method, signals are recorded in the form of a change in voltage over time. The experiment operated with a digital oscilloscope resolution of 16-bit, so at the maximum USB transfer rate, we achieve the highest possible recording frequency of 195 kHz with a recording length of 0.3 s. In total, 3 × 65 Ksamples are recorded. For objective machine assessment of the optimal setup with variations in handle-tip-impact force, it is necessary to select suitable monitored signal parameters. In general, this means a reduction in dimensionality [34], which entails the search for a way to separate representative parameters, the so-called symptoms, from the complex comprehensive information. The term symptom extraction is derived from this. This method is widely used in machine learning. In the field of acoustic NDT, the results of the work of Zhang and colleagues can be mentioned [35]. The mentioned study uses a procedure of feature extraction to create a classification model for the detection of artificially embedded cavities in a reinforced concrete precast. Their algorithm for feature extraction focused on signal decomposition using wavelet decomposition and the calculated energy of the signal and of the frequency spectrum. These values were further supplemented by the value of the dominant frequency, the average frequency and individual spectral moments.

The design of the algorithm in this paper, however, focuses more on the evaluation of qualitative parameters of the signal and frequency spectrum particularly in order to determine the suitability of the used tip-handle-impact force setup. Therefore, we monitor a total of 9 parameters: Dominant frequency *f*_0_, amplitude of the dominant frequency *A*_0_, width of the dominant peak *w*_0_, relative amplitude in relation to the frequency spectrum level *P*_0_, signal energy *E_S_*, signal duration *t_s_*, signal attenuation *A_s_*, attenuation from the frequency spectrum *A_f_* and signal-to-noise ratio (*SNR*).

**Figure 10:**
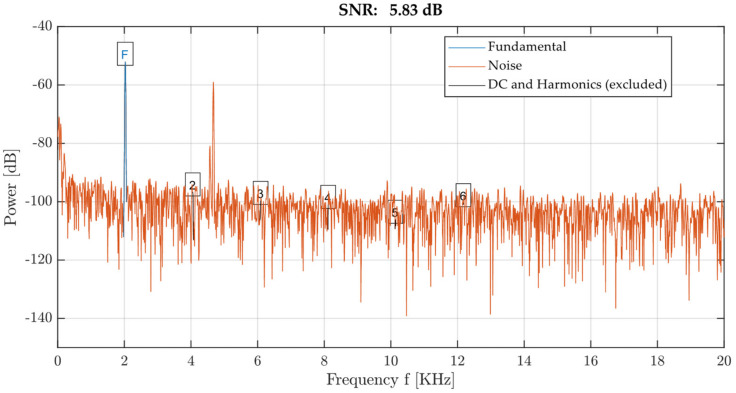
Illustration of signal-to-noise ratio function output.

The individual parameters are shown in Figure 9. The energy of the signal is obtained from integration of the signal above the threshold value T, which is defined as:

(4) $$T=\bar{F}+\left|S_{dt}\right|\times 2$$

where $\bar{F}$ is the average frequency and $\left|S_{dt}\right|$ is the absolute value of the standard deviation of the measured frequency. Signal duration *t_s_* is then defined by the signal area for the values *S* > *T*. Attenuation from the frequency spectrum is the ratio of the amplitude *A*_0_ and the width of the dominant frequency *w*_0_. *SNR* is then obtained using the *snr* function from the Signal Processing Toolbox [36]. An example of this type of evaluation is shown in Figure 10. The signal-to-noise ratio value is given in dB.

### 2.3. Multi-Criteria Signal Evaluation

Each combination of the selected type of hammer, sensor or impact force can result in a different quality of the generating impulse and thus in a different quality of the response of the test specimen. This objective quality can be assessed in terms of readability of the frequency spectrum, signal length, noise level of the recorded signal, etc. Optimisation of these input parameters is often based on a heuristic approach, when the observed response satisfies the requirements of the technician conducting the test, or of the technical legislation governing the test. However, if we seek to objectively evaluate this decision-making process and determine, for example, which combination is optimal for a given technical purpose, the use of the feature extraction technique (mentioned in the previous chapter) and subsequent multi-criteria evaluation of extracted parameters seems adequate.

If it is necessary to unambiguously decide and analytically compare different variants with each other based on n-parameters, we can use the Multiple Criteria Decision Making (MCDM) algorithm and, inter alia, the Analytic Hierarchy Process (AHP), which was developed in 1970 by Saaty [37]. Utilisation of this algorithm can be observed in numerous applications when it is necessary to select the most suitable variant according to the selected weights of partial parameters. An example can be the search for the optimal route for the transfer of oversized loads [38] by the team Wolnowska et al. This procedure was also employed to evaluate the optimal NDT diagnosis method for testing the damage of prestressing units in an extensive study by Hurlebaus et al. [39]. The utilisation of this algorithm is such a sensitive matter as the determination of damage of prestressing units documents the reliability and effectiveness of the AHP algorithm.

Let us assume that we are observing several different parameters on the acquired signals *A*_1_… *A_n_*, for example, the deviation of the dominant frequency from the actual resonant frequency of the tested specimen. This parameter can be designated *f*_1_… *f_n_*. The measured deviations can be used to create a matrix of ratios between partial deviations, by which we compare each individual deviation with all others. This matrix is also called an estimation matrix for a given parameter and can be expressed as follows:

(5) f1f1f1f2⋯f1fnf2f1f2f2⋯f2fn⋮⋮⋱⋮fnf1fnf2⋯fnfn·f1f2⋮fn=n·f1f2⋮fn

We therefore acquire a comparison matrix *A* = ($a_{ij}$), $a_{ij}$ = *f_i_*/*f_j_*, *i*,*j* = 1…*n* that comprises only positive numbers and is governed by the condition:

(6) $$a_{jk}\times a_{kj}=1$$

Each ratio of the parameter *f_i_*/*f_j_* is then a separate weight and expresses the importance of the parameter *f_j_* to *f_i_*. This weight cannot be determined precisely and must be estimated based on the given application. Table 1 presents the recommended weight variances that were defined by Saaty in his theory for adequate setting of the mutual importance of individual monitored parameters.

The resulting comparison matrix is then as follows:

(7) 11w1,2⋯1w1,nw1,21⋯1w2,n⋮⋮1⋮w1,nw2,n⋯1

The product *S_i_* is then acquired for each row and is subsequently used for the calculation of the priority vector *F_i_* of each parameter:

(8) $$S_{i}=\overset{n}{\underset{j=1}{\prod }}S_{ij}$$

(9) $$R_{i}={\left(S_{i}\right)}^{\frac{1}{n}}$$

(10) $$F_{i}=\frac{R_{i}}{\sum_{i=1}^{n}R_{i}}$$

The calculation of the partial weight *b_ij_* of the parameter *f* of the signal *A* can be acquired using the following formula:

(11) $$b_{ij}=\begin{array}{c} MAX\to b_{ij}=\frac{a_{ij}-MIN\left(a_{i}\right)}{MAX\left(a_{i}\right)-MIN\left(a_{i}\right)}\\ MIN\to b_{ij}=\frac{MAX\left(a_{i}\right)-a_{ij}}{MAX\left(a_{i}\right)-MIN\left(a_{i}\right)} \end{array}$$

In this case, it is essential to consider the requirement of a given parameter, where it is necessary to define which values are preferred. For example, when evaluating the deviation of the measured dominant frequency from the actual resonant frequency of the specimen, it is desirable if the deviation reaches the lowest possible values. If we want the resonant frequency to be sufficiently readable, we want the amplitude to reach the highest possible value. Equivalently to the first example, it is also possible to determine the desired amplitude value and to require the lowest absolute deviation from the required value of the measured amplitude.

The partial score of the monitored parameter is then calculated using the formula:

(12) $$a_{ij}=f_{i}\times b_{ij}$$

If we then have *n* number of *A* signals for which we monitor the 9 above-mentioned parameters by expressing the total score of each recorded signal in a different combination of handle/tip/impact force, the final score of each combination is the sum of the partial scores of the monitored parameters.

## 3. Results

### 3.1. Data Acqusition

**Figure 11:**
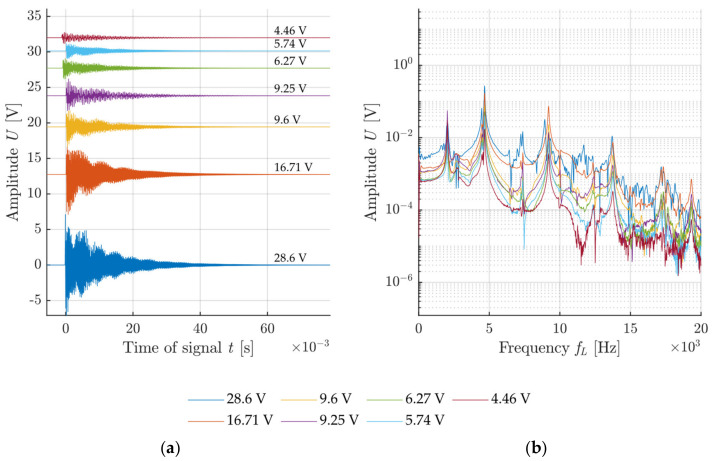
Example of impact with a hammer with a blunt tip without a handle: (**a**) Signals from the sensor attached to the test specimen with rising impacting force; (**b**) frequency spectrum of recorded signals.

**Figure 12:**
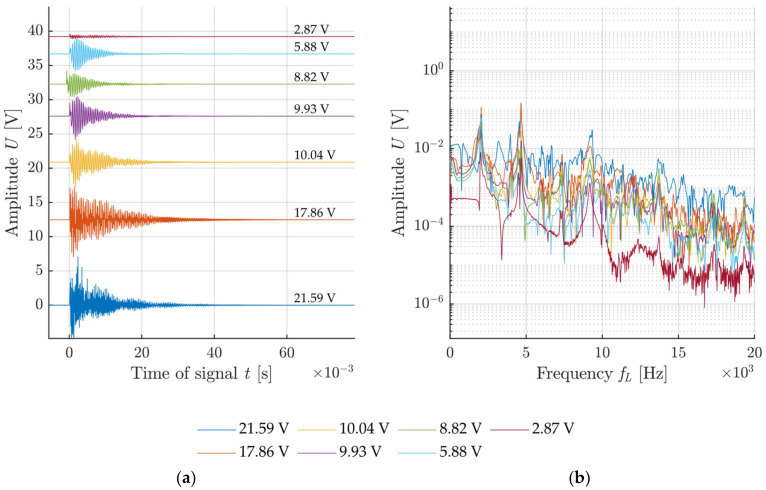
Example of impact with a hammer with a sharp tip without a handle: (**a**) Signals from the sensor attached to test specimen with rising impacting force; (**b**) frequency spectrum of recorded signals.

**Figure 13:**
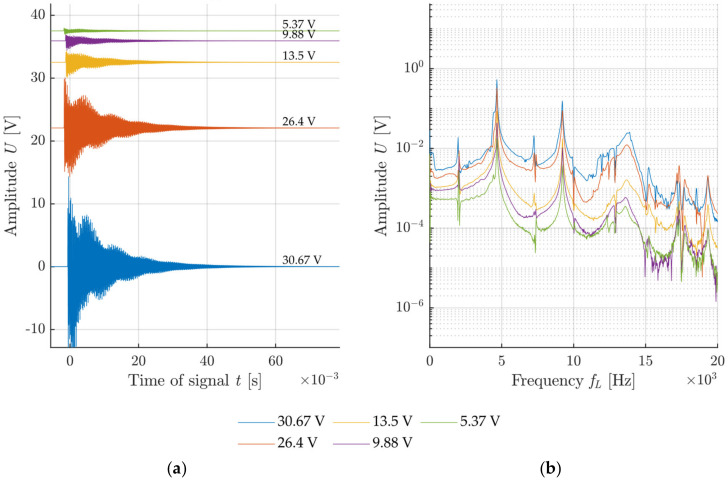
Example of an impact with a hammer with a blunt tip with a handle: (**a**) Signals from the sensor attached to test specimen with rising impacting force; (**b**) frequency spectrum of recorded signals.

**Figure 14:**
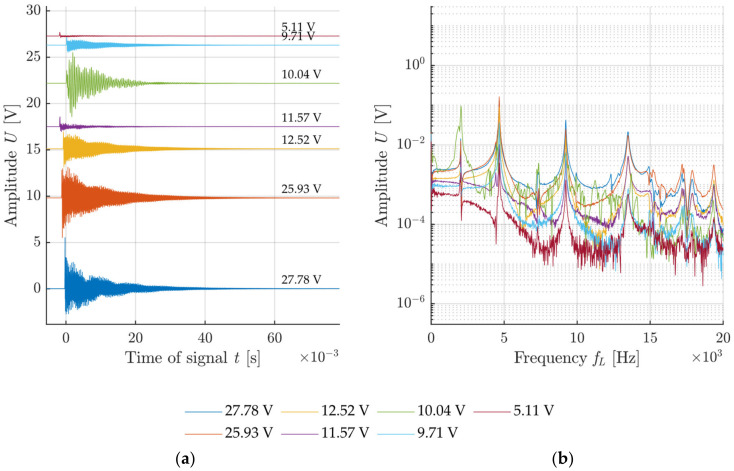
Example of impact with a hammer with a sharp tip with a handle: (**a**) Signals from the sensor attached to test specimen with rising impacting force; (**b**) frequency spectrum of recorded signals.

The voltage generated at the impact was used for the description of the impact intensity for each configuration. The graphs in Figure 11, Figure 12, Figure 13 and Figure 14 show the specimen response to the generated signal in the time and frequency domains processed by the FFT. The left graph is always in the amplitude–time region, where the horizontal axis describes the time t(s) and the vertical axis the measured sensor voltage U(V). The right graph is the frequency spectrum for individual responses, where the horizontal axis describes the frequency *f_L_* (Hz) and the vertical axis describes the measured sensor voltage U(V).

The resonant frequency of the calibration concrete specimen, which was verified in a laboratory, reached 4.6 kHz, which corresponds to the highest-frequency peak in the frequency spectrum. The comparison of the measured results in Figure 11 and Figure 12 indicates a noisier signal when using a sharp hammer tip. Especially in the case of lower intensities, signal noise occurs already from 10 kHz. The increased noise is caused by the crushing of the surface of the test specimen by the sharp tip when the tip slightly penetrates the specimen. This phenomenon does not occur with specimens that are harder than the hammer material.

A comparison of results when using the handle is shown in Figure 13 and Figure 14. Both cases exhibit an evidently higher minimum impact intensity as the handle makes it impossible for the impact to be performed with a very low level of intensity. In addition, substantial noise from the signal can be observed again in the variant with a sharp tip, starting already at 7 kHz. Again, this is caused by disruption of the surface matrix of the concrete specimen.

### 3.2. Feature Extraction and Comparision

A weighted multi-criteria comparison can be used to compare the effectiveness of the used combination of tip-handle. The recording of all the signals presented above was performed on the same test specimen, where the only change involved the handle of the hammer and the tip, and the individual impact impulses were generated by different forces. This approach offers a comparison of all performed impacts with each other and a design of suitable weights of the monitored parameters for the evaluation of each acquired signal.

In this case, the monitored parameters include the dominant frequency, its amplitude, peak width, peak prominence, energy under the curve and signal duration.

All performed measurements therefore included the acquisition of these parameters, which were subsequently assigned a respective weight in relation to each other. As a result, a multi-criteria comparison [37] is particularly effective as it allows us to define which parameters are more or less important for us based on the set weights and simultaneously enables us to qualitatively evaluate each acquired signal. This approach can be seen in the pre-processing of some world publications, as an introductory tool for separating well-acquired signals from signals with various anomalies, which are not suitable for further assessment. The individual set weights of the monitored parameters are presented in Table 2.

When assessing the frequency spectrum, a correctly measured resonant frequency is one of the most important parameters for the operator or technical diagnostician. As illustrated in chapter 1.4, in the case of the frequency spectrum, it is common for the signal to contain a frequency other than the resonant frequency in the given test direction. That is why the weight of the dominant frequency parameter is highest when compared to other monitored parameters. Conversely, the peak width has a lower weight than a correctly measured frequency. The amplitude is important for the decision of which the measured frequency is dominant in the frequency spectrum. It is therefore important for these two parameters to be measured accurately to guarantee correct measurement.

In the presented case, we know the correct frequency of the test specimen, which is exactly 4667 Hz. This parameter is fixed as it is still the same specimen, and the others may differ significantly depending on the character of the impact, the impact force, the shape of the hammer tip, etc.

The proposed ratios of the weights of individual parameters were based on experience with the localisation of frequency peaks. For accurate assessment, it is key that the deviation from the dominant frequency Δ*f*_0_ reaches minimum values. This parameter is important for the recalculation of other material characteristics such as element thickness, velocity of longitudinal waves or dynamic modulus of elasticity of the tested material. If the test specimen is vibrated by a small energy only, there is a chance that the natural frequency of the specimen will not be recognisable in the frequency spectrum, which necessitates the requirement for the highest possible signal energy. However, if the vibrating energy is too high, the acquired signal may suffer from noise, and therefore, a sufficiently low level of noise, expressed by the SNR value, is required. In the case of the proposed weights, a sufficiently high SNR value is more important than the acquired amplitude, signal energy or signal attenuation, which consequently balances out the high amplitude requirement. This approach enables the set weights of the comparison matrix to be used for signals acquired by the resonance method from various constructions.

**Figure 15:**
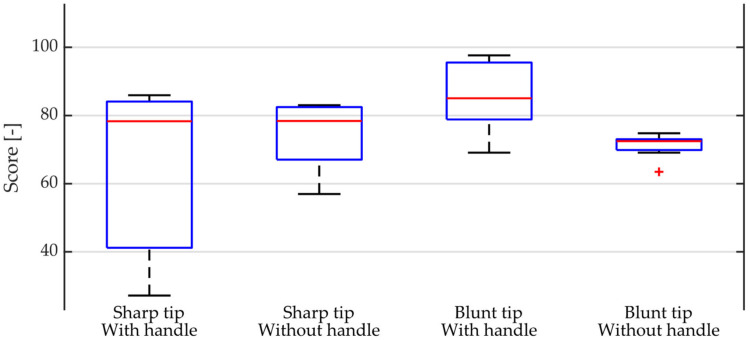
Boxplot of score distribution for each combination of tip and handle.

If we process the performed measurement, we obtain a point evaluation of each performed impact, which includes the evaluation of all the monitored parameters. If we express the average point evaluation in each combination of handle tip, we obtain a point evaluation, which indicates, based on the set parameter weights, that the best results are achieved by the combination of blunt tip and handle. The partial score of the acquired signals can be used to create a box plot, which is shown in Figure 15.

The sharp tip in both variants without a handle/with a handle exhibits a large variance in its evaluation, which indicates that it is more difficult to generate the right intensity and direction of impact with the hammer in this combination. By contrast, the blunt tip with the handle exhibits only a small variance in the signal score between the values 60 and 90. This means it is easier to generate the ideal vibrating impulse with a hammer with a blunt tip and therefore to obtain the ideal response of the test specimen.

### 3.3. Correlation of Extracted Features

The individual monitored signal parameters can also be compared with each other using the correlation coefficient *R_coeff_*. This comparison is presented in Table 3. Expected dependencies between the individual parameters can be observed in this case. The highest correlation is achieved between signal energy and amplitude and prominence. Other significant correlations occur between frequency attenuation and signal energy and the prominence of the dominant frequency peak. The correlation of the signal-to-noise ratio does not value 0.3, which is marked as a weak correlation based on Evans’ study [40]. Despite the fact that this value does not a reach high correlation with the other monitored parameters, it is an important parameter in terms of readability of the frequency spectrum.

The correlation comparison also reveals that the parameters of amplitude *A*_0_ and prominence *P*_0_ are almost interchangeable within the measured signals. Nevertheless, it is important to note that the test specimen whose resonant frequency was measured was in good condition and it can therefore be assumed that the frequency spectrum will reach a very low noise level. However, if the measurement were performed on a test specimen that had already been degraded (presence of cracks, exposure to frost or high temperatures, etc.), it can be expected that the value of the dominant frequency amplitude *A*_0_ will reach values other than the prominence *P*_0_ of the dominant frequency.

## 4. Discussion

The assessment of the signal acquired during vibroacoustic testing is an important task that usually takes place at the manual level. The analysis of the acquired signals disregards signals that lack the required information or which are not sufficiently readable. This pre-processing is essential in automated data processing and therefore plays an important part in the design of ever-evolving applications in the field of machine learning. The proposed procedure utilises the technique of the extraction of specific symptoms in the time and frequency domains, which are subsequently evaluated by a multi-criteria comparison using Saaty’s methods.

The proposed procedure was applied to monitor the influence of the used impact hammer, the used tip, handle and impact force on the quality of the measured signal using a piezoelectric sensor during standard impact-echo testing.

The test specimen with the dimensions 100 × 100 × 400 mm was produced from standard plain C40/45 concrete and was kept in a water bath under laboratory conditions after formwork removal.

The proposed procedure allowed us to assess the acquired acoustic signals and determine whether they contain the required information such as resonant frequency, amplitude, signal energy, frequency attenuation and signal attenuation. The procedure is valuable in the case of the automated processing of measured signals and their evaluation, which enables the establishment of a threshold value of the signal score, which then contains a sufficient amount of information to be included in further assessment.

The proposed procedure can be effectively applied to a series of measurements which are expected to exhibit oscillation of the natural resonant frequency in the specified range. The procedure was verified in the frequency range from 0.5 Hz to 20 kHz. Its use can also be applied to ultrasonic measurements, although those measurements require slightly different parameters, such as the velocity of ultrasound in a material. However, the proposed procedure approaches signals without the inclusion of the frequency domain.

In the case of technical applications, hundreds of signals are often acquired, whether they pertain to measurements of test specimens or measurements of test points on larger structures such as walls, bridges or roads. If we accept that some measurements in these applications can be conducted under sub-optimal conditions and exhibit a type of system or random error in regard to the generation of natural vibrations, it is desirable to have an automated procedure that will detect such erroneously measured signals. Otherwise, the measurement may provide completely incorrect information and will therefore constitute a false-positive error. Furthermore, this procedure adds the possibility to qualitatively assess the conducted measurement and determine, for example, how successful the measurement was with regard to different aspects (type of impact, sampling frequency, homogeneity of generated pulses, position of generating impacts, location of the placed sensors, etc.).

## 5. Conclusions and Future Work

The presented paper’s main motivation is our current evaluation of IE measurement, which heavily relies on the ‘manual’ approach. These results should act as a reference for the measurement of real-life damaged test specimens or structures. Our future work will focus on a case study to verify the designed algorithm as a tool to evaluate its accuracy and ability to distinguish different characteristics of the measurement setup and acquired signals itself. It is much harder to record the pulse-echo signal of deteriorated building materials. Such a material has different attenuation and different acoustic impedance with hand with different physical-mechanical properties such as density, compressive strength and modulus of elasticity. The designed algorithm was used for simple pulse-echo testing of the concrete beam under laboratory conditions but present a minimal functional example, considered as the reference state. Future testing should focus on concrete of different mixtures, age, water saturation and initial degradation state.

## Figures and Tables

**Table 1:** Saaty’s scale [37].

Value:	1	3	5	7	9	(2,4,6,8)
Importance:	Same importance	Slight importance	Significant importance	Very significant importance	Absolute importance	Intermediate Values

**Table 2:** Weights set for signal classification of each hammer–handle setup.

Parameters	*A*_0_	Δ*f*_0_ *	*w*_0_	*P*_0_	*E_S_*	*t_s_*	*A_s_*	*A_f_*	*SNR*	Demand	*Fi*
*A*_0_	1.00	0.20	0.33	0.50	0.50	0.50	0.50	0.50	1.00	Max	0.05
Δ*f*_0_ *	9.00	1.00	0.50	0.33	0.33	1.00	1.00	1.00	3.00	Min	0.12
*w*_0_	3.00	2.00	1.00	0.20	0.17	0.50	0.33	0.20	2.00	Min	0.07
*P*_0_	3.00	3.00	5.00	1.00	0.50	0.17	1.00	1.00	0.20	Max	0.10
*E_S_*	2.00	3.00	6.00	2.00	1.00	1.00	1.00	1.00	0.33	Max	0.14
*t_s_*	2.00	1.00	2.00	6.00	1.00	1.00	1.00	0.17	0.25	Max	0.10
*A_s_*	2.00	1.00	3.00	1.00	1.00	1.00	1.00	0.33	0.50	Min	0.10
*A_f_*	2.00	1.00	5.00	1.00	1.00	6.00	3.00	1.00	2.00	Max	0.19
*SNR*	1.00	0.33	0.50	5.00	3.00	4.00	2.00	0.50	1.00	Max	0.12

* Difference between actual resonant frequency of specimen and localised dominant frequency.

**Table 3:** Correlation table of all extracted features of signals.

Parameters	*A*_0_	Δ*f*_0_ *	*w*_0_	*P*_0_	*E_S_*	*t_s_*	*A_s_*	*A_f_*	*SNR*
*A*_0_	1.000	-	-	-	-	-	-	-	-
Δ*f*_0_ *	0.296	1.000	-	-	-	-	-	-	-
*w*_0_	−0.168	−0.800	1.000	-	-	-	-	-	-
*P*_0_	1.000	0.297	−0.169	1.000	-	-	-	-	-
*E_S_*	0.994	0.288	−0.154	0.994	1.000	-	-	-	-
*t_s_*	0.214	−0.319	0.414	0.213	0.208	1.000	-	-	-
*A_s_*	−0.117	0.311	−0.269	−0.116	−0.117	−0.773	1.000	-	-
*A_f_*	0.985	0.409	−0.299	0.986	0.975	0.157	−0.077	1.000	-
*SNR*	0.243	−0.164	0.281	0.243	0.207	0.209	−0.036	0.211	1.000

* Difference between actual resonant frequency of specimen and localised dominant frequency.

## Data Availability

The data presented in this study are openly available in FigShare at DOI: doi.org/10.6084/m9.figshare.13653284.v3.
